# Serum uric acid and male erectile dysfunction: A Mendelian randomization study

**DOI:** 10.1097/MD.0000000000044578

**Published:** 2025-09-12

**Authors:** Wenyu Chi, Weihui Jia, Chengrong Zhang, Guobao Sun

**Affiliations:** a Department of Urology, Affiliated Hospital of Shandong Second Medical University, Weifang, Shandong Province, China; b Department of Gastroenterology, Weifang People’s Hospital, Weifang, Shandong Province, China.

**Keywords:** causation, correlation, erectile dysfunction, Mendelian randomization, serum uric acid

## Abstract

To evaluate the potential causal relationship between serum uric acid levels and erectile dysfunction in men, this study performed a Mendelian randomization (MR) analysis. The exposure factor was serum uric acid, and the outcome factor was erectile dysfunction. All data come from genome-wide association studies, and 2-sample MR studies are conducted based on R Studio 4.3.2 and the “TwoSampleMR” R software package. The inverse-variance weighted and MR-Egger methods are mainly used for analysis. Neither the inverse-variance weighted (Beta = 0.068; SE = 0.062; *P* = .274) nor the MR-Egger (Beta = 0.079; SE = 0.101; *P* = .436) approaches demonstrated a causal association between the 2 variables. According to this MR study, there is no causal relationship between serum uric acid levels and male erectile dysfunction, so medication to lower uric acid levels alone may not be effective in treating erectile dysfunction in patients with high serum uric acid.

## 1. Introduction

Erectile dysfunction (ED) in males refers to the inability to achieve or sustain an erection of the penis that is enough for effective vaginal intercourse. This condition is prevalent among middle-aged and elderly men.^[1]^ Recent studies indicate that various causes contribute to the development of ED, including psychological, neurological, hormonal, vascular, drug-related, and a combination of multiple factors.^[2]^ Vascular factors are the most common among these, including atherosclerosis, hypertension, hyperlipidemia, smoking, diabetes, and pelvic radiotherapy. Endothelial dysfunction is a common mechanism underlying many vascular risk factors that contribute to arterial ED.^[3]^ Endothelial cells create nitric oxide (NO), which is crucial for the erection process. NO can activate soluble guanylyl cyclase, causing an increase in guanosine 3′,5′-cyclic monophosphate. This, in turn, causes cytoplasmic calcium depletion and smooth muscle relaxation in the cavernous region.^[4]^

Uric acid (UA) is a weak acid produced by purine metabolism that is mostly present in the form of urate at physiological pH.^[5]^ Serum UA can stimulate vascular smooth muscle cells to proliferate and produce a large amount of angiotensin II. This causes oxidative stress and glycocalyx loss, which reduces nitric oxide activity in blood vessels, boosts inflammatory cytokine levels, and ultimately impairs the function of vascular endothelial cells.^[6]^ Serum UA levels were shown to be positively associated with vasculogenic ED in a previous case-control study.^[7]^ In addition, an observational systematic review and meta-analysis concluded that serum UA is an independent risk factor for male erectile dysfunction.^[8]^ However, current research on UA and ED are retrospective in nature, making them subject to confounding factors such as a small sample size, a short follow-up period, and the combined effect of other factors on erectile dysfunction.

Mendelian randomization (MR) uses an instrumental variable to imitate an individual’s random allocation of exposure factors. The instrumental variable is a single nucleotide polymorphism (SNP). During the creation of human gametes, the allele of a specific SNP is randomly assigned to egg or sperm cells. This also accounts for the influence of confounding factors on the exposure–outcome causal link, reducing concerns about reverse causation.^[9]^ This work employs MR to clarify the causal link between UA and ED, resulting in novel clinical approaches for ED treatment and prevention.

## 2. Methods

### 2.1. Study design and data sources

**Figure 1.**
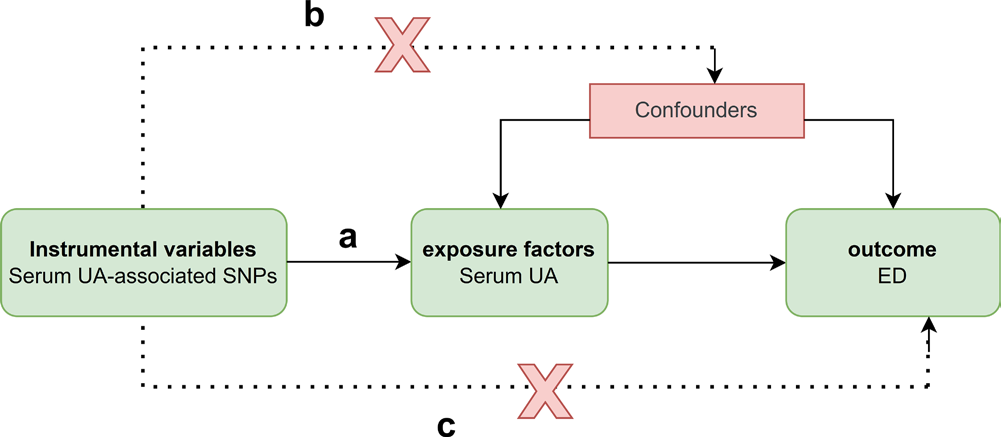
Schematic diagram of the Mendelian randomization method between serum uric acid levels and erectile dysfunction. (A) Genetic variation directly affects exposure factors. (B) Genetic variation is not associated with any confounders. (C) Genetic variation affects outcome only through exposure factors. Dashed lines indicate potential causal relationships that violate Mendelian randomization principles.

The instrumental variable chosen using Mendelian randomization must meet the following 3 conditions (Fig. 1).^[10]^ First, the genetic variation of the instrumental variable is inextricably linked to the exposure factors. Second, genetic variation has no relationship with other confounding factors. Third, genetic variation can only be influenced by risk factors. This is the sole way to influence the outcome and cannot be achieved in any other way.

The data for this study were derived from genome-wide association studies (GWAS). The sample size of the dataset for SNPs associated with serum uric acid was 129,405, including 12,499,459 SNPs, which was derived from Sakaue S et al.^[11]^ The sample size of the dataset for SNPs associated with male erectile dysfunction was 223,805, including 9310,196 SNPs, which was derived from Bovijn J et al.^[12]^ Table 1 summarizes the basic information from both databases. We investigated SNPs linked to serum UA. The first phase involved retaining SNPs with genome-wide significance (*P* < 5 × 10^−8^) and removing SNPs in linkage disequilibrium (*r*^2^ threshold = 0.001 and clumping distance = 10,000 kb). The second stage is to remove SNPs related with confounding factors. We examined the human genotype–phenotype association database (PhenoScanner V2). The third step involves excluding weak instrumental factors, such as SNPs with an *F*-statistic < 10. We did data harmonization to ensure that the SNP’s effect on exposure and outcome corresponded to the same allele. Finally, 59 SNPs were used in the Mendelian randomization analysis. There is no need for extra ethical evaluation for this work because the GWAS database is publically available and the initial study was approved by the ethics review committee.

**Table 1:** Basic information on serum UA and ED groups in the study.

Exposure	Population	Year	Sample size	Data sources	Web source
Serum UA	East Asian	2021	129,405	Sakaue S et al^[11]^	https://gwas.mrcieu.ac.uk/datasets/ebi-a-GCST90018757/
ED	European	2018	223,805	Bovijn J et al^[12]^	https://gwas.mrcieu.ac.uk/datasets/ebi-a-GCST006956/

ED = erectile dysfunction, UA = uric acid.

### 2.2. Statistical analysis

This study used 5 2-sample MR analysis methods: inverse-variance weighted (IVW), MR-Egger, weighted median, simple mode, and weighted mode. The major analytical methods are IVW and MR-Egger. IVW is a weighted linear regression method that combines the effects of multiple instrumental variables (e.g., genetic variants). It assumes that all instrumental variables meet the 3 core MR assumptions: relevance (associated with the exposure), independence (not confounded by external factors), and exclusion restriction (affects the outcome only via the exposure). IVW is the most efficient method when these assumptions hold and is well-suited for estimating the overall causal effect. Unlike IVW, MR-Egger regression accounts for horizontal pleiotropy, where instrumental variables may influence the outcome through pathways other than the exposure. The intercept term in MR-Egger regression is used to detect and correct for unbalanced pleiotropy, adding robustness to the analysis. These 2 methods complement each other. IVW provides efficient causal effect estimates under strict assumptions, while MR-Egger offers a safeguard against pleiotropic bias, making the results more robust.

In sensitivity analysis, Cochran *Q* test was utilized to examine heterogeneity of MR analysis. This test evaluates heterogeneity in the instrumental variable effects. If heterogeneity is significant (*P* < .05), a random-effects model is applied to account for variability; otherwise, a fixed-effects model is used for greater precision. To evaluate if genetic pleiotropy influences the causal relationship, the MR-Egger regression intercept is used. The intercept term in MR-Egger regression assesses whether unbalanced pleiotropy might bias the causal inference. A significant intercept indicates pleiotropic effects. The leave-one-out analysis was done to determine the stability of the results. This approach iteratively removes 1 instrumental variable at a time and reanalyzes the data to assess whether the results are driven by any single instrument. These analyses enhance the robustness and reliability of the study by addressing potential biases and ensuring the results are not unduly influenced by individual instrumental variables or pleiotropy.

This study utilized R Studio version 4.3.2 (Posit, PBC, Boston) and the “TwoSampleMR” R software package to conduct MR analysis. A *P*-value of <.05 was deemed statistically significant.

## 3. Results

### 3.1. Two-sample Mendelian randomization results

**Figure 2.**
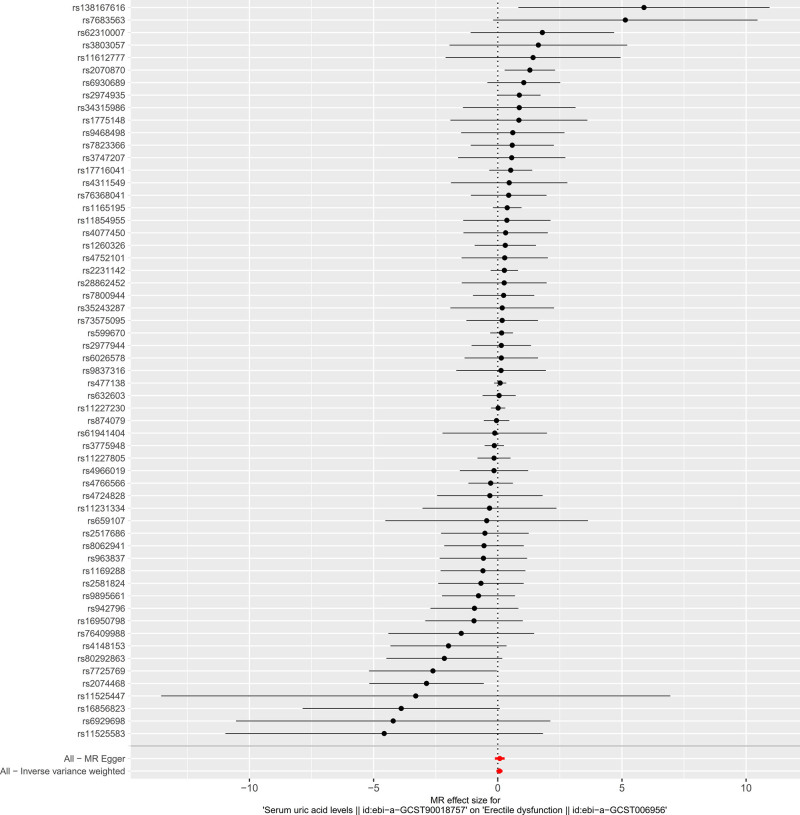
Forest plot of single SNPs associated with serum UA and ED. Each point represents an effect size, with horizontal lines indicating 95% confidence intervals. The red points show the combined estimates using inverse-variance weighted and MR-Egger methods. The vertical dashed line represents the null hypothesis of no causal effect. ED = erectile dysfunction, UA = uric acid.

The results of Mendelian randomization between serum UA levels and ED are shown in Table 2. All 5 MR methods showed no causal association between serum UA and ED (all beta values are >0, and all *P* values are >.05). The forest plot of individual SNPs associated with serum UA and ED is shown in Figure 2. Among these 59 SNPs, the 95% confidence intervals (CIs) of most SNPs span the middle invalid line (odds ratio = 0). After combining the effect sizes, the 95% CIs for the IVW and MR-Egger Mendelian randomization methods straddle the middle null line (odds ratio = 0). The lower limit of the 95% CI for the IVW method is 0.947, and the upper limit is 1.210. The lower limit of the 95% CI for the MR-Egger method is 0.888, and the upper limit is 1.320.

**Table 2:** MR estimates for each method of assessing the causal effect of serum UA on ED.

MR method	Number of SNPs	Beta	SE	*P* value	OR	Lower limit of 95% CI	Upper limit of 95% CI
MR-Egger	59	0.079	0.101	.436	1.083	0.888	1.320
Weighted median	59	0.072	0.086	.407	1.074	0.907	1.273
IVW	59	0.068	0.062	.274	1.071	0.947	1.210
Simple mode	59	0.195	0.149	.196	1.215	0.907	1.628
Weighted mode	59	0.053	0.072	.468	1.054	0.915	1.214

ED = erectile dysfunction, UA = uric acid.

**Figure 3.**
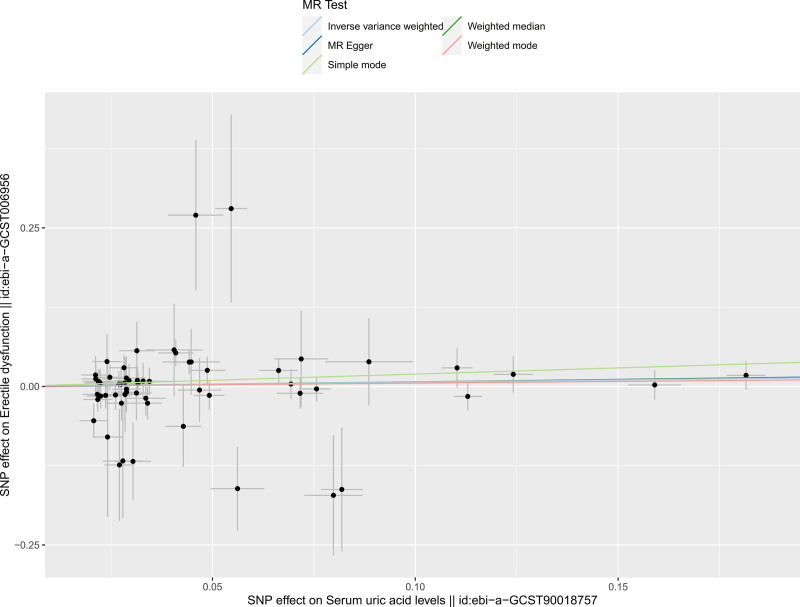
Scatter plot of correlation between serum UA and ED. Each point represents the effect size of individual genetic variants, with the fitted line showing the overall trend. The dashed line indicates the null hypothesis of no effect. ED = erectile dysfunction, UA = uric acid.

The scatter plot of the correlation between serum UA and ED is shown in Figure 3. The *x*-axis is the impact of SNP on serum UA, and the *y*-axis is the impact of SNP on ED. Each point in the scatter plot represents each SNP, and the line passing through each point represents the 95% CI. The vertical coordinate of each point is the effect of SNP on ED, and the horizontal coordinate is the effect of SNP on serum UA. The ratio of the 2 effects is the slope. The straight-line slopes of these 5 MR methods in the scatter plot are close, and the slope is slightly >0. Combined with the results of the MR analysis, the correlation between serum UA and ED is weak (*P* > .05).

### 3.2. Sensitivity analysis

**Figure 4.**
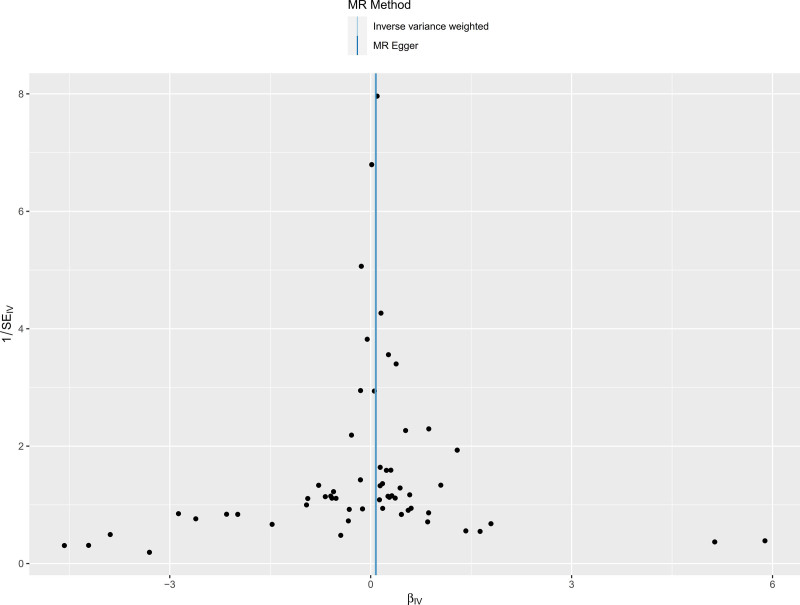
Funnel plot to assess heterogeneity of MR analysis. Each point represents the effect size after excluding 1 genetic variant at a time, with confidence intervals shown. The dashed line indicates the overall MR estimate, highlighting the robustness of the findings.

**Figure 5.**
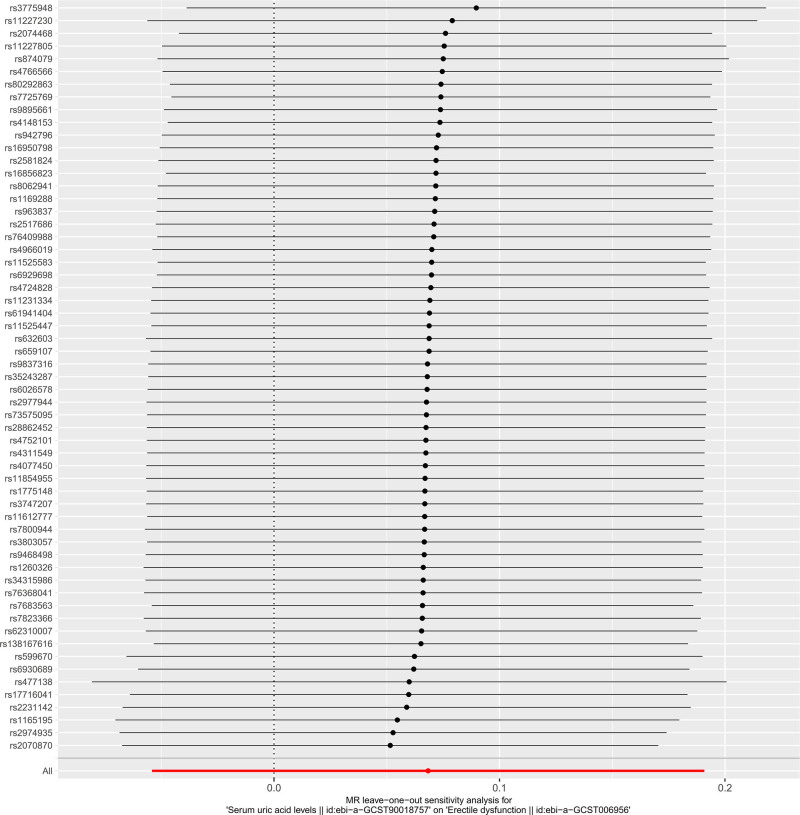
Leave-one-out forest plot. Each point represents an individual genetic variant, with asymmetry indicating potential bias. The dashed vertical line represents the overall MR estimate.

Cochran *Q* test was used to conduct heterogeneity analysis on the results after MR analysis by the MR-Egger method and the IVW method. As shown in Table 3, there is no heterogeneity in the MR analysis of the 2 main methods (*P* < .05). In the funnel plot in Figure 3, SNPs are distributed roughly symmetrically on both sides of the IVW and Mr. Egger straight lines. The intercept of MR-Egger regression was used to evaluate horizontal pleiotropy in MR analysis. The results are shown in Figure 4. The intercept is −0.000809, the standard error is 0.00582, and the *P* value is 0.890, indicating that there is no horizontal pleiotropy. The leave-one-out method is used to evaluate the reliability and stability of the results. As shown in Figure 5, after excluding each SNP, the overall error does not change much, indicating that the results are reliable.

**Table 3:** Heterogeneity analysis results of Cochran *Q* test.

Method	*Q*-statistics	Degree of freedom	*P* value
MR-Egger	62.401	57	.291
IVW	62.422	58	.322

IVW = inverse-variance weighted.

## 4. Discussion

This is the first study to use a 2-sample Mendelian randomization strategy to investigate the causal link between serum UA and ED. The data indicate that there is no causal relationship between serum UA and ED. In the MR sensitivity analysis, there was no heterogeneity or horizontal pleiotropy. MR analysis was done after 1 SNP was removed at a time, and the results changed less, indicating that the results were steady and reliable. Of course, the 2 are just genetically related and have no causative relationship, but it is possible that serum UA and ED are linked at other levels outside genetics.

Multiple prior systematic reviews and meta-analyses have found that greater serum uric acid levels are related with the development of ED.^[8,13,14]^ Furthermore, clinical prediction model studies have demonstrated that serum UA can be used as a risk factor for ED.^[15]^ Furthermore, hyperuricemia may be regarded an independent risk factor in addition to known risk factors. This is completely different from the findings of this study, and we feel there are primarily the following reasons: First, all previous research were observational, with no randomization, prospective, or blinding procedures. Differences in study results could be attributed to the limits of observational studies, which are prone to confounding factors and reverse causality. Second, the populations included in these studies may be complicated by other vascular illnesses, such as atherosclerosis, hypertension, and other confounding variables, reducing the trustworthiness of the findings. Interestingly, however, in a multicenter cross-sectional investigation, UA was identified as an independent protective factor for ED, which contradicts the findings of this study.^[16]^ Because of the limitations of cross-sectional studies, causality cannot be established; however, there is a correlation between serum UA levels and ED. A cross-sectional investigation in Finland found no apparent risk relation between serum UA levels and ED, which supports the findings of this study.^[17]^ As a result, the association between UA levels and ED remains hotly debated. This study simply confirmed at the genetic level that there is no causal link between serum UA and ED.

Although a lot of case-control studies and meta-analyses have been conducted, the underlying biological mechanism between serum UA and ED remains unknown and requires additional investigation. Serum uric acid contains both pro-oxidative and antioxidant properties in the body. Roumeliotis S et al believe that hyperuricemia-induced oxidative stress may contribute to endothelial cell senescence and death.^[18]^ Oxidative stress is caused by an imbalance between pro-oxidant and antioxidant components, which stimulates the production of pro-oxidants. It is assumed that xanthine oxidase produces a large amount of superoxide, causing smooth muscle cells to produce lectin-type oxidized LDL receptor 1, which harms endothelial cells and reduces NO production. Less NO causes cells to produce less cyclic guanosine monophosphate, which is detrimental to smooth muscle and blood flow in the penis’ corpus cavernosum.^[4,19]^ This study demonstrates that there is no genetic relationship between serum UA and ED, implying that several prior observational studies may have been influenced by other confounding factors, resulting in false-positive results. De Becker B et al investigated serum uric acid levels and related oxidative stress markers and discovered that serum UA had no significant relationship with oxidative stress.^[20]^ This suggests that serum UA affects endothelial cells not just through oxidative stress, but also via additional routes. Masi S et al observed a U-shaped association between uric acid levels in the blood and microvascular remodeling characteristics such as the media-to-lumen ratio (M/L ratio). They also discovered that both low and high amounts of uric acid in the blood were associated with hypertrophic remodeling of tiny resistance arteries.^[21]^ Serum uric acid is a strong antioxidant at physiological levels and may protect vascular endothelial cells. However, low levels of serum UA, particularly in the extracellular fluid, may drastically reduce the antioxidant ability. High amounts, on the other hand, promote its mobility within cells, triggering pathways for cell proliferation and pro-oxidative inflammation.^[22]^ This study’s Mendelian randomization analysis implies a linear relationship between serum UA and ED, which may not be the case. It has previously been proposed that damage to the vascular endothelium is a possible biological explanation for ED caused by high serum UA levels. Masi S et al hypothesized that low serum UA levels could cause endothelial problems, which contradicted the findings of several earlier research. Because observational epidemiological research make it difficult to eliminate biases such as confounding factors and reverse causality, etiological interpretation is limited. As a result, the mechanism by which blood uric acid levels affect vascular endothelial cells has to be investigated further.

This study offers the following benefits: First, there are currently no MR studies that have investigated the causative relationship between serum UA and ED, and the majority of them are observational. MR analysis is a superior statistical method that eliminates the effects of confounding variables and reverse causation. Second, we employ the IVW and MR-Egger methods as primary assessment methods, with weighted median, simple mode, and weighted mode as secondary evaluation methods, to assure the results’ stability and reliability. Third, the summary statistics generated from GWAS in this study have bigger sample sizes than observational studies, implying that valid causal inferences have more statistical strength.

Of course, this study does have some limitations. First, our study sample was limited to those of European and East Asian heritage; future research is needed to look into other populations. Second, SNPs related to exposure variables and SNPs related to outcomes are from distinct ethnic groups, resulting in a population stratification bias due to disparities in allele frequencies and disease prevalence between ethnic groups. Third, given the *F* statistics were all >10, this study should be free of weak instrumental variable bias; however, the poor power could be attributed to the small number of SNPs utilized as instrumental variables. Fourth, while our analysis used a large population-based cohort, we acknowledge that statistical power may differ between genetic variations and outcomes. Fifth, we have a poor understanding of the molecular mechanisms that relate genetic variation to exposure, which may influence how results are interpreted.

## 5. Conclusion

This MR study shows that there is no causal relationship between serum uric acid levels and male erectile dysfunction. Therefore, in patients with high serum uric acid, simply using medication to reduce uric acid levels may not be effective in treating erectile dysfunction.

## Acknowledgments

We are grateful to the IEU Open GWAS project for their cooperation with data.

## Author contributions

**Formal analysis:** Weihui Jia.

**Methodology:** Weihui Jia.

**Resources:** Chengrong Zhang.

**Software:** Wenyu Chi.

**Visualization:** Chengrong Zhang.

**Writing – original draft:** Wenyu Chi.

**Writing – review & editing:** Guobao Sun.
